# Design of silver-zinc-nickel spinel-ferrite mesoporous silica as a powerful and simply separable adsorbent for some textile dye removal

**DOI:** 10.1038/s41598-024-66457-4

**Published:** 2024-07-17

**Authors:** Ehab A. Okba, Moamen F. Rabea, Mohamed Y. El-Sheikh, Eman F. Aboelfetoh

**Affiliations:** Chemistry Department, Faculty of Science, Tanta University, Tanta, 31527 Egypt

**Keywords:** Spinel ferrite@SiO_2_, Cationic and anionic dyes, Adsorption, Wastewater treatment, Pollution remediation, Environmental chemistry, Physical chemistry, Surface chemistry

## Abstract

Silver-zinc-nickel spinel ferrite was prepared by the co-precipitation procedure with the precise composition Ag_0.1_Zn_0.4_Ni_0.5_Fe_2_O_4_ for bolstering pollutant removal effectiveness while upholding magnetic properties and then coated with a mesoporous silica layer. The surface characteristics and composition of Ag_0.1_Zn_0.4_Ni_0.5_Fe_2_O_4_@mSiO_2_ were confirmed using EDX, FT-IR, VSM, XRD, TEM, SEM, and BET methods. The surface modification of Ag-Zn-Ni ferrite with a silica layer improves the texture properties, where the specific surface area and average pore size of the spinel ferrite rose to 180 m^2^/g and 3.15 nm, respectively. The prepared spinel ferrite@mSiO_2_ has been utilized as an efficient adsorbent for eliminating methyl green (MG) and indigo carmine (IC) as models of cationic and anionic dyes from wastewater, respectively. Studying pH, Pzc, adsorbent dosage, dye concentration, and temperature showed that efficient removal of MG was carried out in alkaline media (pH = 12), while the acid medium (pH = 2) was effective for IC removal. Langmuir isotherm and pseudo-second-order kinetics were found to be good fits for the adsorption data. Both dyes were adsorbed in a spontaneous, endothermic process. A possible mechanism for dye removal has been proposed. The adsorbent was effectively recovered and reused.

## Introduction

The rapid industrialization of recent times has resulted in a significant escalation in the release of diverse toxic materials into our marine environment, presenting a critical hazard to environmental preservation^1^. The textile industry is one of the anthropogenic activities that most consume water and pollute water bodies^2^. Synthetic dyes, which originate from the chemical and textile sectors, are particularly worrisome among these pollutants on account of their enduring nature, toxicological properties, and ability to cause environmental damage^3^. The removal of these harmful dye pollutants has emerged as a critical area of research, given the dual implications for safeguarding human health and the environment.

Adsorption is commonly used to remove organic pollutants and dyes from wastewater. Although there are many methods for treating wastewater, including oxidation, flocculation, coagulation, membrane filtration, chemical precipitation, ion exchange, and biological treatment^4–7^, adsorption is widely regarded as a straightforward, low-cost, risk-free, and effective method for removing dyes. Bentonite^8^, clay minerals^9^ and agricultural waste-based adsorbents^10^ are only a few of the adsorbents that have been used for dye removal from aqueous solutions.

One effective method for removing dye is by using magnetic materials, specifically metal ferrites. Due to their magnetic properties, metal ferrites have shown significant potential for absorbing and eliminating different dye compounds from wastewater. Metal ferrites possess distinctive attributes, including elevated surface area, heightened magnetic susceptibility, and chemical stability, which make them well-suited contenders for environmental cleanup^11–13^. Nevertheless, improving the efficiency of mixed ferrites for the purpose of dye removal continues to be a difficult task, but it can be overcome by implementing surface modification techniques.

Metal ferrites possess the desirable characteristic of being adjustable, which greatly enhances their appeal for a wide range of uses such as catalysis^14^, sensors^15^, dielectric^16^, biomedical^17^, and environmental remediation^18^. Due to their magnetic properties, these substances may be easily separated from the treated water, making them very suitable for magnetic-based systems designed to remove dyes^19^.

Numerous variables influence the properties of ferrite, such as cation distribution^20,21^, preparation conditions, size, microstructure, thermal treatment, and synthesis method. Ferrites have been produced through a variety of processes^21–23^. Co-precipitation is regarded as an exceptional synthesis technique owing to its numerous advantages, which encompass minute particle size^24^, exceptional homogeneity, and the possibility of producing crystalline and high-yield nanoparticles of ultrafine size may be produced via this process^25^. Spinel ferrites have become a significant group of ferrites that belong to the metal ferrite family. These compounds exhibit a cubic crystal structure where magnetic cations are evenly distributed in octahedral and tetrahedral positions^26^, leading to their remarkable magnetic characteristics^27^.

Nickel-ferrite nanoparticles NiFe_2_O_4_ is a spinel ferrite with high magnetic, chemical, optical, electrical, and dielectric properties^28,29^. Which is used for adsorption and other application^18,30^. Zn^2+^ and Ag^+^ ions were often selected among various dopants. Zinc ferrites are ordinary spinel ferrite with chemical, optical, electrical, and dielectric properties. There are numerous potential applications for zinc ferrites, including hyperthermia medication for cancer^31^, photocatalysis^32^, organic transformation catalysts^33^, wastewater remediation^34^, and sensors^35^. The incorporation of silver into metal ferrite nanoparticles has shown significant promise in enhancing different elements of wastewater treatment^36,37^. The addition of silver improves the adsorption efficiency of magnetic ferrite by introducing more active sites on its surface and enhances its photocatalytic degradation and antibacterial properties^38,39^. As a result, these composite materials are highly efficient at removing pollutants and ensuring the quality and safety of water resources.

Although mixed ferrites possess inherent characteristics that make them effective for dye removal, there is still potential for enhancing their efficiency and selectivity. This is when the importance of silica coating becomes relevant. Silica is a favorable material for surface coating due to its stability, chemical inertness, lack of toxicity, large surface area, high rigidity, thermal stability, low cost, being environmentally friendly, and facile modification^40–42^. Silica coating improves the adsorption capacity over a wide range of pH values, which makes it suitable for adsorption under varying solution conditions^43^. The thermal stability of the silica layer allows for regeneration and reuse of the adsorbent after adsorption cycles. This versatility makes silica coating suitable for a wide range of adsorption applications.

In the current study, a novel silver-zinc-nickel ferrite is synthesized and coated with mesoporous silica Ag_0.1_Zn_0.4_Ni_0.5_Fe_2_O_4_@mSiO_2_ as a powerful adsorbent to remove methyl green (MG) and indigo carmine (IC) as models of cationic and anionic dyes, respectively, from wastewater.

## Experimental

### Chemical reagents

**Figure 1:**
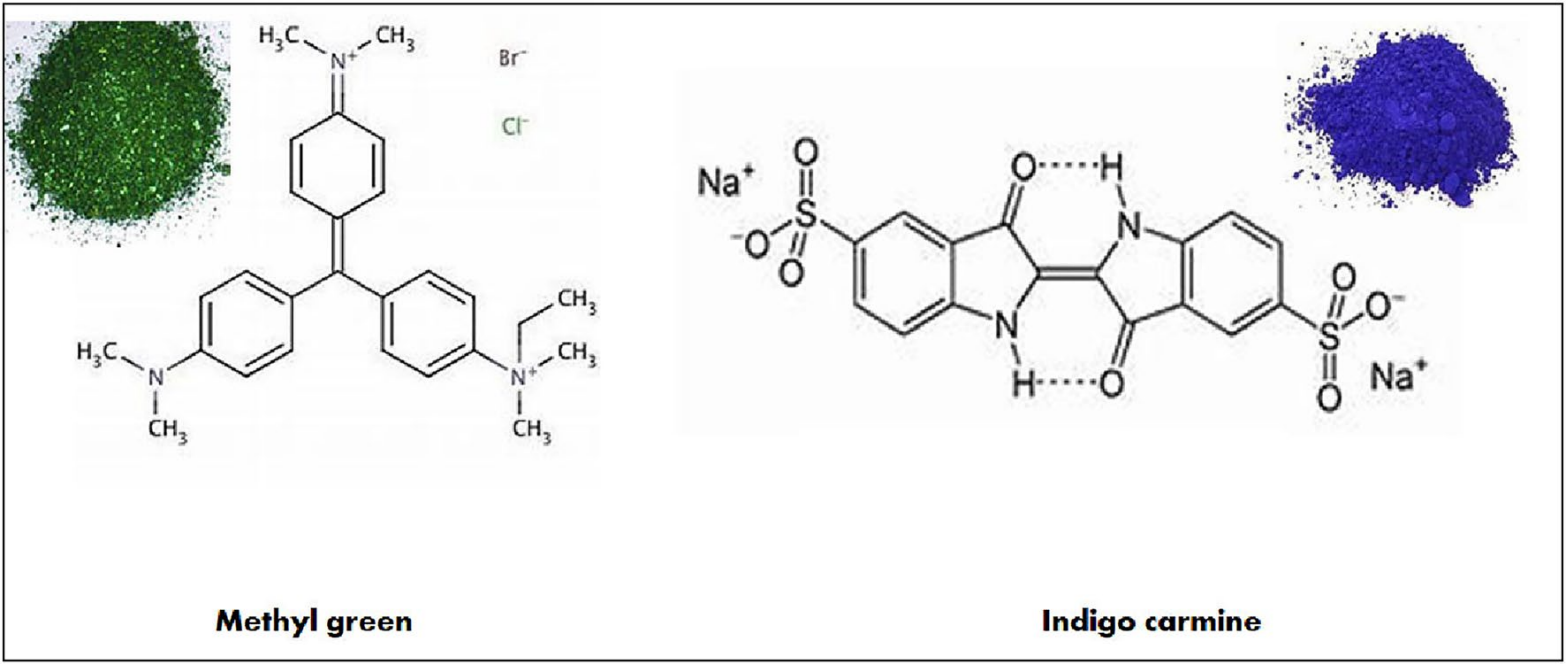
Structure of the investigated dyes.

Pure and high grade ferric nitrate nonahydrate (Fe (NO_3_)_3_.9H_2_O) (98%), zinc nitrate hexahydrate (Zn (NO_3_)_2_.6H_2_O) (98%), nickel nitrate hexahydrate (Ni(NO_3_)_2_.6H_2_O) (97%), silver nitrate (AgNO_3_) (99%), cetyl trimethyl ammonium bromide (CTAB) (98%), tetraethyl ortho silicate (TEOS)(98%) were obtained from Sigma-Aldrich chemicals, methyl green (C_27_H_35_BrClN_3_) (85%) and indigo carmine (C₁₆H₈N₂Na₂O₈S₂) (85%) were obtained from Merck, and Fig. 1 showed their molecular structures.

### Synthesis of spinel ferrite (SF)

Mixed ferrite with different compositions, Ag_x_Zn_(0.5-x)_Ni_0.5_Fe_2_O_4,_ where x = (0.1, 0.3, 0.5), was prepared by the co-precipitation method^44^. Briefly, Zn (NO_3_)_2_.6H_2_O (0.4 M), AgNO_3_ (0.1 M), Ni (NO_3_)_2_.6H_2_O (0.5 M), and Fe (NO_3_)_3_.9H_2_O (2 M) were dissolved in distilled water and mixed together with a molar ratio of 1:2, and then NaOH (3 M) was added until a precipitate was formed. The reaction was refluxed at 80°C for two hours. The brownish precipitate of spinel ferrite was then magnetically decanted. The precipitate was washed with distilled water and then ethanol. The sample was dried overnight at 80°C and then characterized using a variety of analytical techniques.

### Synthesis of spinel ferrite@mSiO_2_(SFS)

**Figure 2:**
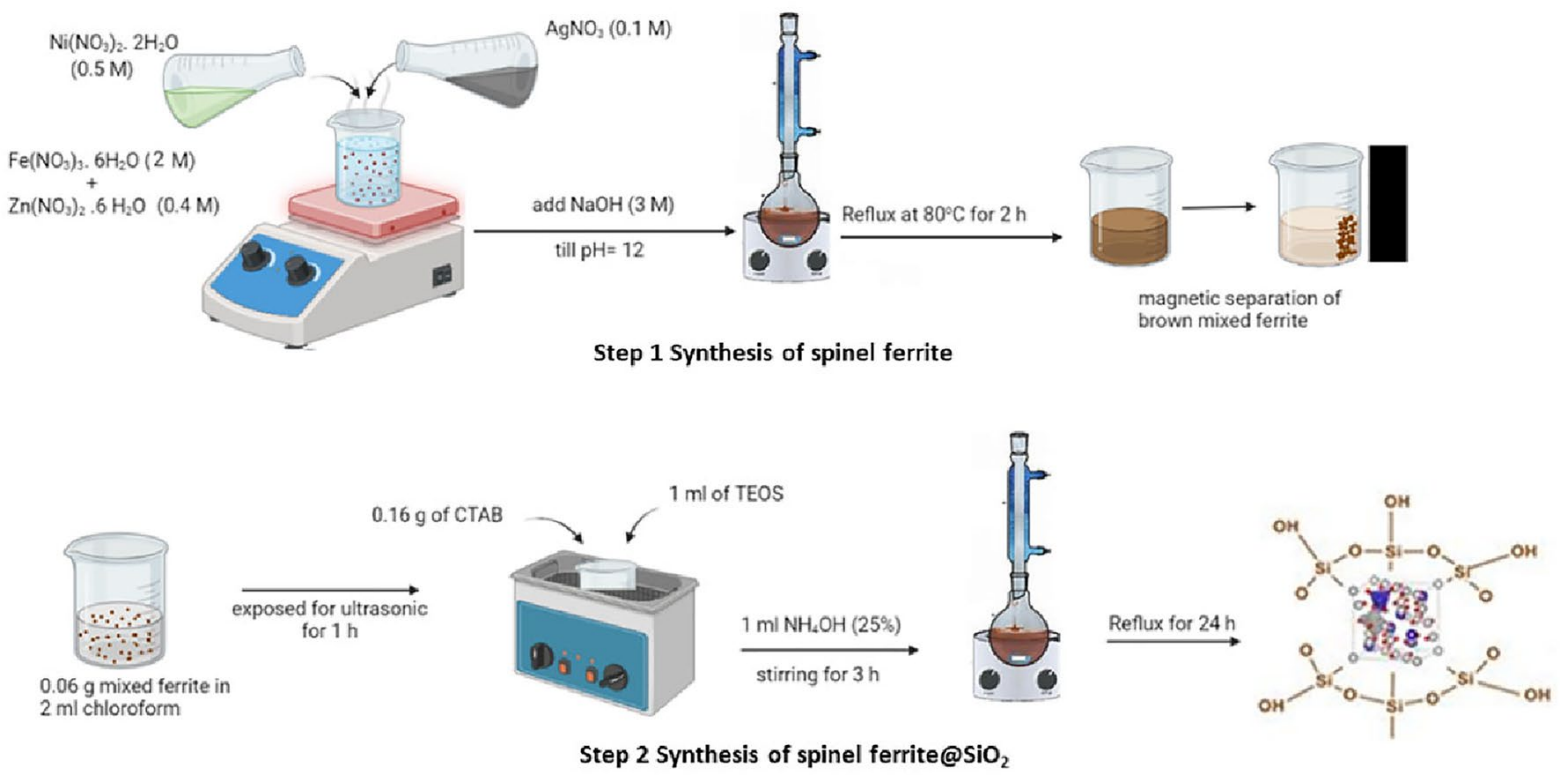
Illustrative steps for the synthesis of SFS.

Ag_0.1_Zn_0.4_Ni_0.5_Fe_2_O_4_@mSiO_2_ (SFS) was synthesized according to Liu et al^45^ with modifications as shown in Scheme 2: 0.06 g of previously prepared mixed ferrite and 2 mL of chloroform were mixed and exposed to ultrasonic radiation of frequency 20 kHz (power of 70 W) for 60 min. Then add a mixture of 36 ml ethanol and 25 ml of dist. water to the solution, stirring for 10 min at 30 °C. After dispersing the solution, 0.16 g of CTAB was added and swirled at 65 °C for 30 min. The mixture was agitated for three hours after TEOS (1 ml) and 25% NH_4_OH (1 mL) were added. The gathered product, which is a light brown colour, is rinsed with distilled water and dried for 18 h. The product is removed from CTAB by being refluxed in 200 cc ethanol for 24 h, as shown in Fig. 2.

### Characterization techniques

Powder X-ray diffractometry (XRD) was used to examine the crystalline structure of the prepared samples (GNR APD 2000 PRO diffractometer, CuK α radiation, operated at 40 kV, 30 mA, and 2θ in the range of 10–70°). The molecular structure was studied using FTIR (JASCO, model 4100) with KBr pellets. The elemental compositions were investigated using SEM connected with EDX (IT100LA) operating at 20 kV. The magnetic properties of the samples were measured using VSM (Lake Shore, model 7410). SEM (SU8000 Type II, Hitachi) was used to analyse surface morphology. TEM (JEOL, model JEM-2100) analysis was used to determine particle size and shape. Surface area was determined by the BET method (Quantachrome Touch Win™, version 1.21), and both metal ferrite and metal ferrite@silica were analyzed. Prior to the measurements, the samples were degassed for 12 h at 120 °C. The BJH (Barrett-Joyner-Halenda) method was used to analyse pore size distribution curves and derive the pore volume and pore size (diameter).

### Pzc investigation

To investigate how pH affects spinel ferrite (SF) adsorption, the pH of zero charge (pH_pzc_) was investigated. Rivera et al.^46^, report adjusting the initial pH of 50 ml of 0.01 M NaCl solutions in Erlenmeyer flasks by adding 0.1 M HCl or 0.1 M NaOH solutions to a pH range of 2–12. 0.1 g of adsorbent was added to each flask. After 24 h of agitation at room temperature, the pH of the resulting solutions was determined. By contrasting the initial and final pH values (not shown), the pH_PZC_ was calculated^46,47^. The obtained pH_PZC_ was 6.80 and 6.45 for Ag_0.1_Zn_0.4_Ni_0.5_Fe_2_O_4_ (SF) and Ag_0.1_Zn_0.4_Ni_0.5_Fe_2_O_4_@mSiO_2_ (SFS), respectively.

### Adsorption experiments (batch methods)

To assess the adsorption capabilities of the investigated ferrite, a variety of adsorption experiments have been performed. In each experiment, several 100-mL Erlenmeyer flasks were filled with a known amount of adsorbent (5–30 mg) and 25 mL of a dye solution with a known concentration. The containers were placed in a water shaker thermostat and agitated at 120 rpm at a working temperature. After specified intervals of time, the adsorbent was extracted magnetically. The concentrations of MG and IC in the supernatant were determined by measuring the absorbance at 632 nm for MG and 610 nm for IC by using UV–Vis spectrophotometry (Varian Cary 400). The following Eqs. (1–3) were utilized to determine the dye removal percentage and the amount of dye adsorbed at time t (q_t_) and at equilibrium (q_e_)^48^ $.$

1 $$\text{The dye removal \%}=\frac{{\text{C}}_{\text{o}}-{\text{C}}_{\text{t}}}{{\text{C}}_{\text{o}}} \times 100$$

2 $${q}_{t}=\frac{\left({C}_{o}-{C}_{t}\right)}{m} \times V$$

3 $${q}_{e}=\frac{\left({C}_{o}-{C}_{e}\right)}{m} \times V$$

where C_o_ is the initial concentration, C_t_ is the concentration at time t, and C_e_ is the concentration of the dyes at equilibrium. The volume of the working solution is denoted by V (L), and the mass of the nanocomposite is m (g). Using universal buffer and either HCl (0.1 M) or NaOH (0.1 M), the pH of the solution was maintained within the desired range (2–12).

## Results and discussion

### Characterization

#### XRD

**Figure 3:**
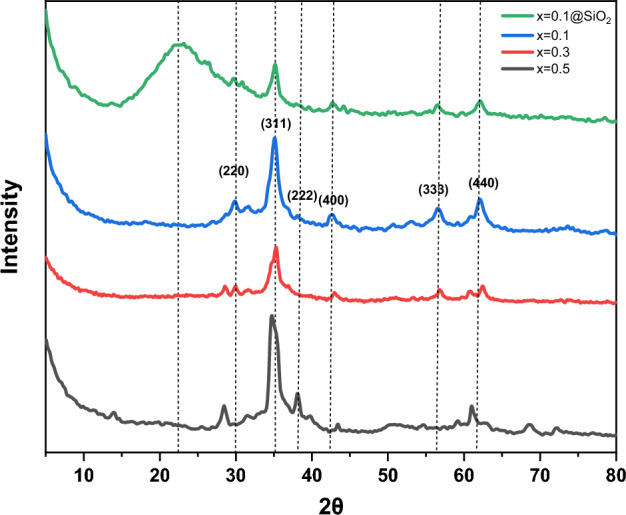
XRD pattern of spinel ferrite Ag_x_Zn_(0.5-x)_Ni_0.5_Fe_2_O_4_ where x = (0.1, 0.3, 0.5) and Ag_0.1_Zn_0.4_ Ni_0.5_Fe_2_O_4_@mSiO_2_.

The phase composition and crystallinity of spinel ferrite Ag_x_Zn_(0.5-x)_Ni_0.5_Fe_2_O_4,_ where x = (0.1, 0.3, 0.5) (SF) and Ag_0.1_Zn_0.4_Ni_0.5_Fe_2_O_4_@mSiO_2_ (SFS) were analyzed by XRD (Fig. 3). All prepared ferrite Ag_x_Zn_(0.5-x)_Ni_0.5_Fe_2_O_4_ exhibited characteristic peaks of the spinel structure of the face-centered cubic (fcc), according to the database of JCPDS (code 00-052-0279)^49^. These peaks appeared at 2θ = 29.8°, 35.1°, 38.07°, 42.65°, 53.09°, 56.56°, and 62.09°, which could be imputed to (220), (3 1 1), (2 2 2), (4 0 0), (4 2 2), (333), and (4 4 0), respectively. The spinel phase’s development is shown by the strong peak visible at the (311) plane^50^. The highest peaks slightly migrated towards lower angles as the Ag^+^ content rose (when x = 0.5), confirming the Ag^+^ substitution for Zn^2+^. Additionally, the peaks widened, indicating that the ferrite nanostructure had formed^51^. The broad peak in the range from 20° to 25° is attributed to the existence of amorphous SiO_2_ in the coated layer^52^.

Scherrer's formula^53^, was used for calculating the size of the crystallite (d) from the strongest peak.

4 $$d=\frac{0.9\lambda }{\beta \text{cos}\theta }$$

where β is the full width at half-maximum for the (311) peak in radians, λ is wavelength, and θ is diffraction angle. The size of the crystallites increased from 5.6 to 10 nm when the Ag^+^ content rose (Table 1). The difference in ionic radii between Ag^+^ (1.26 A°) and Zn^2+^ (0.74 A°) may be the cause of this. It was found that the average crystallite size of silica-coated ferrite increased from 5.6 nm for uncoated ferrite to 8.35 nm. This shows that the silica layer was successfully coated.

**Table 1:** The crystallite size (d) of the prepared sample.

Sample, Ag_x_Zn_(0.5-x)_Ni_0.5_Fe_2_O_4_	Crystallite size D (nm)
X = 0.1@mSiO_2_	8.35
X = 0.1	5.6
X = 0.3	6.5
X = 0.5	10.4

#### FTIR

**Figure 4:**
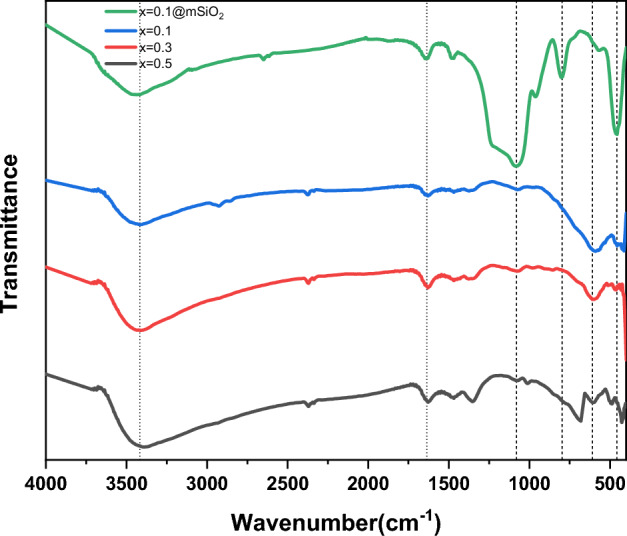
FT-IR spectra of spinel ferrite Ag_x_Zn_(0.5-x)_Ni_0.5_Fe_2_O_4_ where x = (0.1, 0.3, 0.5) and Ag_0.1_Zn_0.4_ Ni_0.5_Fe_2_O_4_@mSiO_2_.

FTIR spectra of prepared metal ferrites are shown in Fig. 4; they span 400–4000 cm^−1^. The distinctive band of all metal ferrite Ag_x_Zn_(0.5-x)_Ni_0.5_Fe_2_O_4_, where x = (0.1, 0.3, 0.5)_,_ are nearly similar, indicating the same structure of all prepared metal ferrite. There are two prominent absorption peaks observed at 585 cm^−1^ and 410 cm^−1^. These peaks correspond to the vibrational modes of tetrahedral metal–oxygen bonds and octahedral metal–oxygen bonds, respectively^54^. Moreover, when the silver doping ratio is increased, particularly at x = 0.5, both the bands related to octahedral and tetrahedral sites display a displacement towards higher wavenumbers. This shift happens due to the replacement of Ag^+^ ions with Zn^2+^ ions, which have a higher atomic weight^55^.

The strong band at approximately 3432 cm^−1^ and the small band at 1638 cm^−1^ refer to the stretching and bending vibrations of OH groups^56^. The spectrum of Ag_0.1_Zn_0.4_Ni_0.5_Fe_2_O_4_@mSiO_2_ (Fig. 4) confirms the presence of the same bands as in mixed ferrite as well as new bands at 1079.54 cm^−1^, 965.52 cm^−1^, 798.08 cm^−1^ and 455.8 cm^−1^. The stretching Si–O–Si, bending Si–O–Si, streching Si–O, and bending Si–O bands are presented here in the correct order^57^. All these bands indicate that SiO_2_ was successfully loaded onto Ag_0.1_Zn_0.4_Ni_0.5_Fe_2_O_4_.

#### EDX

**Figure 5:**
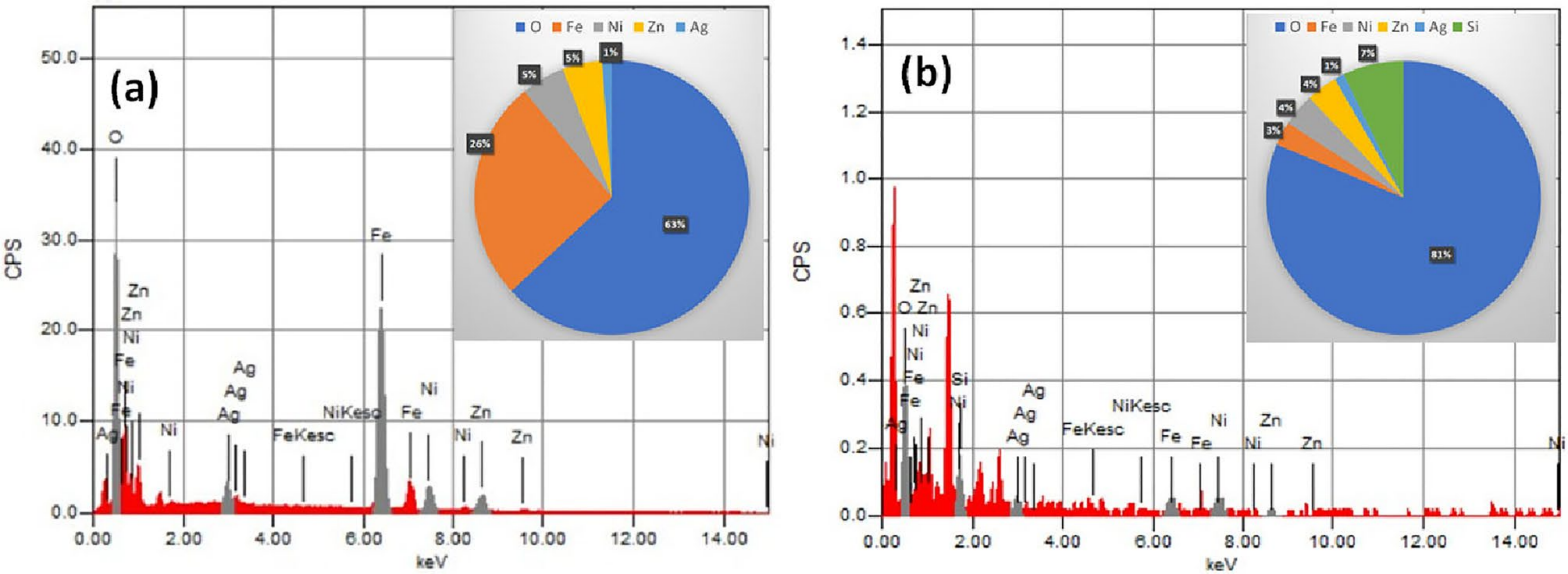
The EDX spectra and atomic percentage of uncoated spinel ferrite Ag_0.1_Zn_0.4_Ni_0.5_Fe_2_O_4_ (**a**) and Ag_0.1_Zn_0.4_Ni_0.5_Fe_2_O_4_@mSiO_2_ (**b**).

EDX was used to analyse synthetic samples for elemental makeup (Fig. 5). The atomic percentages of Ag, Zn, and Ni in addition to Fe and O components in prepared samples indicate that spinel ferrite has been successfully prepared. The main elements of the all spinel ferrite Ag_x_Zn_(0.5-x)_Ni_0.5_Fe_2_O_4_, where x = (0.1, 0.3, 0.5), were Ag, Zn, Ni, Fe, and O (Fig. 5a and Table 2). In addition to the previously mentioned elements, Si appeared in the spectrum of Ag_0.1_Zn_0.4_Ni_0.5_Fe_2_O_4_@mSiO_2_ (Fig. 5b and Table 2), which is largely related to SiO_2_. This reflects the effective loading of silica on the ferrite surface of the composition Ag_0.1_Zn_0.4_Ni_0.5_Fe_2_O_4_.

**Table 2:** The atomic percentage of various samples.

Ag_x_Zn_(0.5-x)_Ni_0.5_Fe_2_O_4_	O %	Fe%	Ni%	Ag%	Zn%
X = 0.1	63.04	25.98	5.15	1.17	4.66
X = 0.3	63.73	27.74	5.87	3.23	2.43
X = 0.5	64.11	25.06	5.14	5.69	0.00

#### VSM

**Figure 6:**
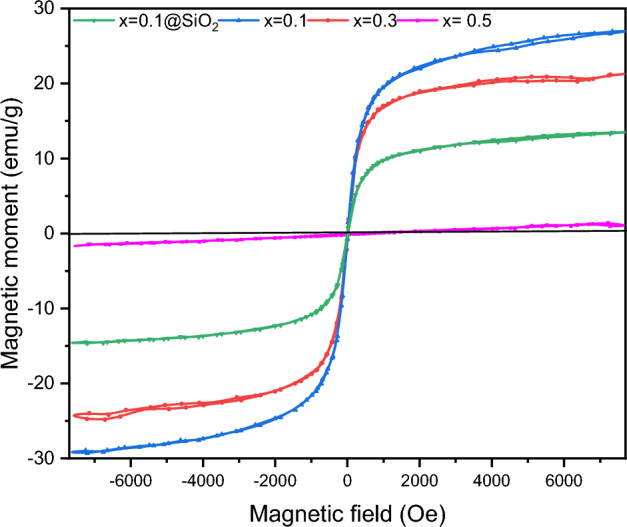
Magnetization curves of spinel ferrite Ag_x_ Zn_(0.5-x)_Ni_0.5_Fe_2_O_4_ where x = (0.1, 0.3, 0.5) and Ag_0.1_Zn_0.4_ Ni_0.5_Fe_2_O_4_@mSiO_2_.

At room temperature, VSM confirmed the samples' magnetic characteristics. The magnetic permeability profiles are shown in Fig. 6. All the different compositions of mixed ferrite Ag_x_Zn_(0.5-x)_Ni_0.5_Fe_2_O_4_, where x = (0.1, 0.3, 0.5), are superparamagnetic, as evidenced by the presence of a slight hysteresis. In Table 3, the values for saturation magnetization, remanence, and coercivity are recorded.

**Table 3:** Saturation magnetization (Ms), remanence magnetization (Mr), and coercivity (H_C_) values.

Sample, Ag_x_ Zn_(0.5-x)_Ni_0.5_Fe_2_O_4_	M_S_(emu/g)	M_r_ (emu/g)	Hc (Oe)
X = 0.1	27.3	0.222	3.628
X = 0.1@mSiO_2_	14.1	0.112	3.54
X = 0.3	21.65	0.218	3.9
X = 0.5	1.5	0.12	12.58

The saturation magnetization values of mixed ferrite Ag_x_Zn_(0.5-x)_Ni_0.5_Fe_2_O_4_, where x = (0.1, 0.3, 0.5), decrease as the ratio of Ag increases, mainly due to the presence of diamagnetic silver components in the Ag-based nanoferrite samples^58,59^. In the case of Ag_0.1_Zn_0.4_Ni_0.5_Fe_2_O_4_@mSiO_2_, the nonmagnetic silica shell is responsible for the drop in saturation magnetization, coercivity, and remanence of Ag_0.1_Zn_0.4_Ni_0.5_Fe_2_O_4_@mSiO_2_^60,61^. However, the saturation magnetization of 14.1 emu/g was sufficient for facile separation of Ag_0.1_Zn_0.4_Ni_0.5_Fe_2_O_4_@mSiO_2_ from aqueous solutions with a magnet.

#### SEM and TEM

**Figure 7:**
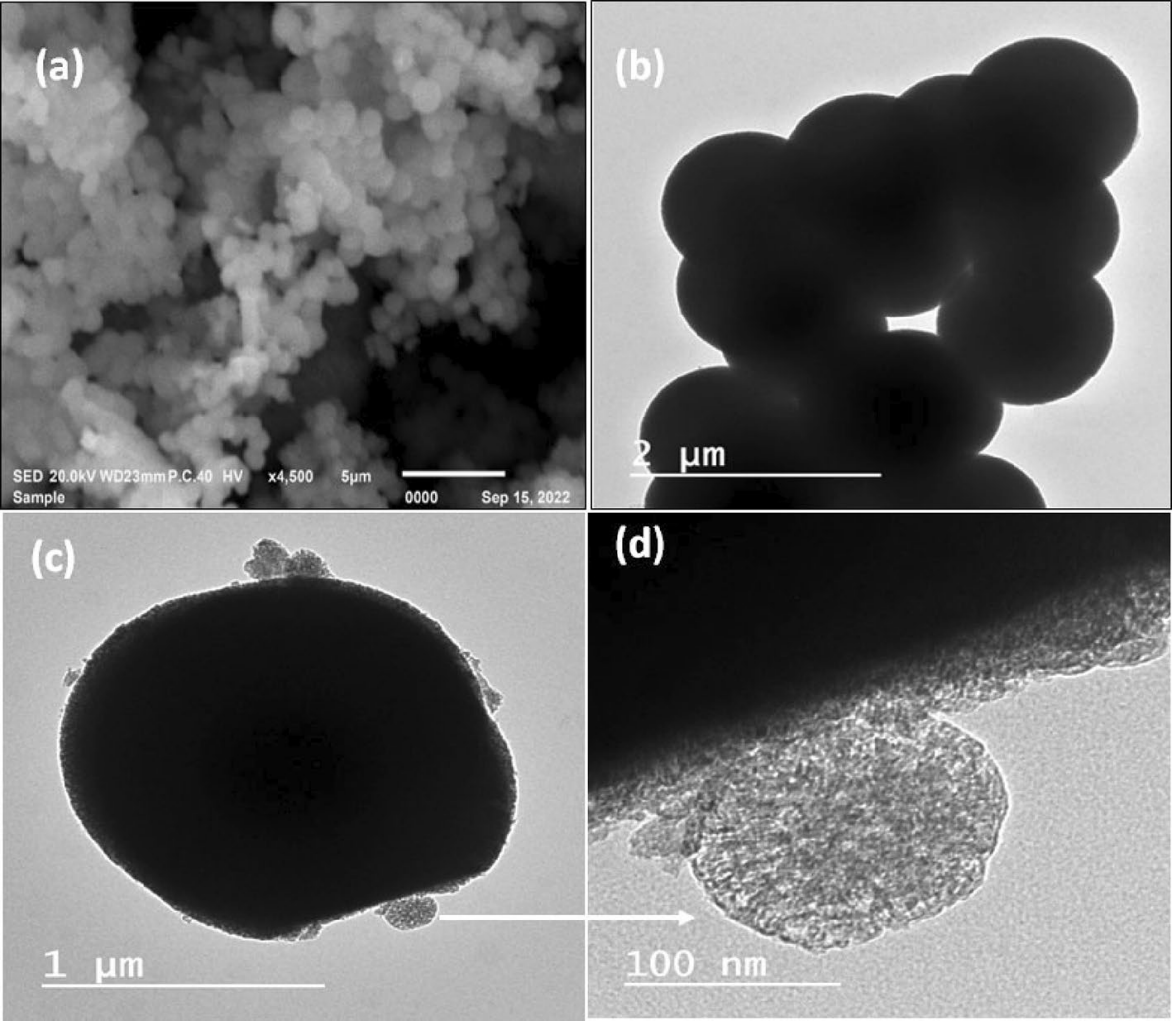
SEM image of SFS (**a**) and TEM images of SF (**b**) and SFS (**c**,**d**).

SEM and TEM techniques were employed to examine the morphological characteristics of the samples. In the SEM image of Ag_0.1_Zn_0.4_Ni_0.5_Fe_2_O_4_@mSiO_2_ (Fig. 7a), a smooth surface composed of spherical crystals was observed. TEM images of Ag_0.1_Zn_0.4_Ni_0.5_Fe_2_O_4_ and Ag_0.1_Zn_0.4_Ni_0.5_Fe_2_O_4_@mSiO_2_ illustrated the spherical morphology of the particles, with an average particle size of approximately 1 µm (Fig. 7b,c). Figure 7c exhibited the successful encapsulation of the ferrite within a thin core–shell structure of silica. Additionally, Fig. 7d displayed the presence of a thin mesoporous silica layer with silica aggregations at various locations on the surface of the ferrite, highlighting its porous nature in HRTEM.

#### Textural characteristics

**Figure 8:**
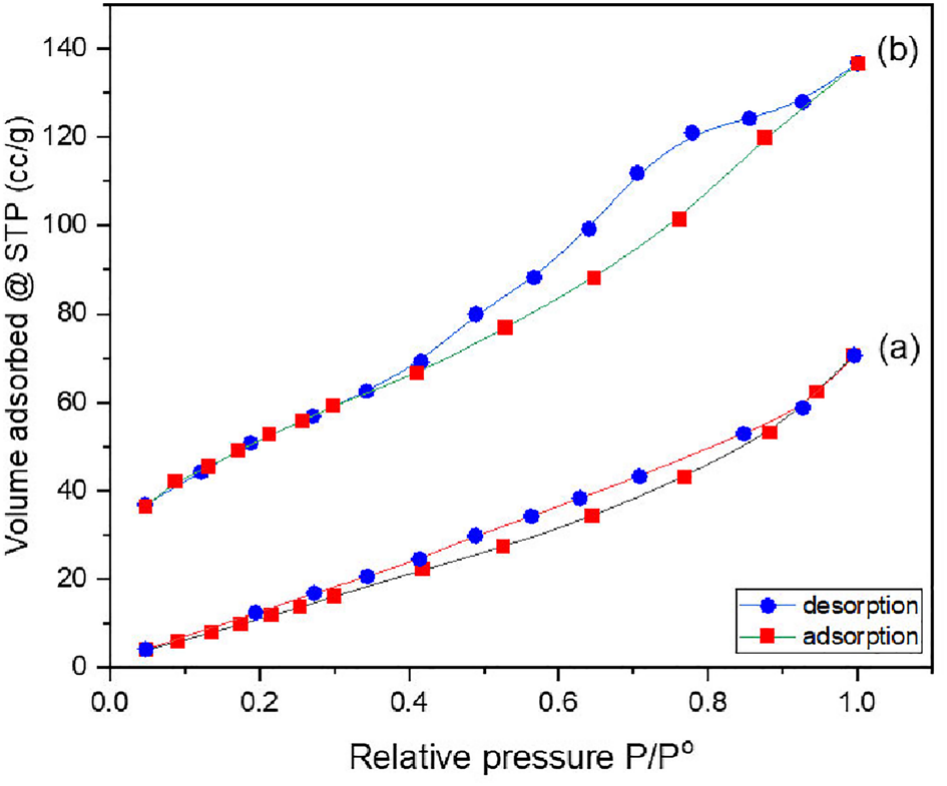
Adsorption–desorption of N_2_ gas by SF (**a**) and SFS (**b**).

The adsorbent surface area is a key factor in raising adsorption efficiency. According to BET and porosity measurements, the specific surface area and pore volume of spinel ferrite were determined to be 69.79 m^2^/g and 0.404 cm^3^/g, respectively. Coating the prepared spinel ferrite with a silica layer increased the specific surface area and pore volume to 180.81 m^2^/g and 0.771 cm^3^/g, respectively. The synthesized materials are porous, as shown by the average pore size (Table 4). According to N_2_-adsorption/desorption isotherm Fig. 8, both the SF and the SFS displayed a type IV with a hysteresis loop of the H_2_(b) type, indicating a mesoporous nature with a wide range of pore size distribution^62^. This takes place in porous materials characterized by a network of interconnected pores as well as in pores characterized by a broad body and a thin neck^63,64^.

**Table 4:** The data of surface and pore properties.

Adsorbent	BET (m^2^/g)	Average pore radius (nm)	Total pore volume (cc/g)
SF	69.79	2.32	0.404
SFS	180.81	3.15	0.771

### Adsorption studies

**Figure 9:**
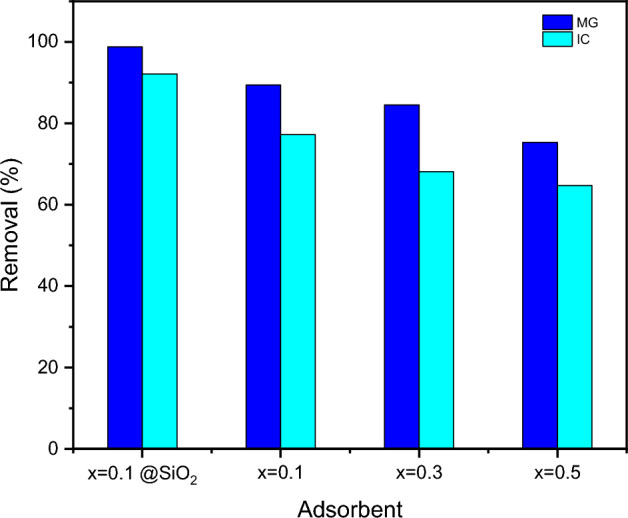
The comparison of the removal efficiency of MG (4 mg/L) and IC (19 mg/L) utilising 20 mg of various adsorbents Ag_x_Zn(_0.5−x)_Ni_0.5_Fe_2_O_4_ at pH = 7 and 25 °C.

**Figure 10:**
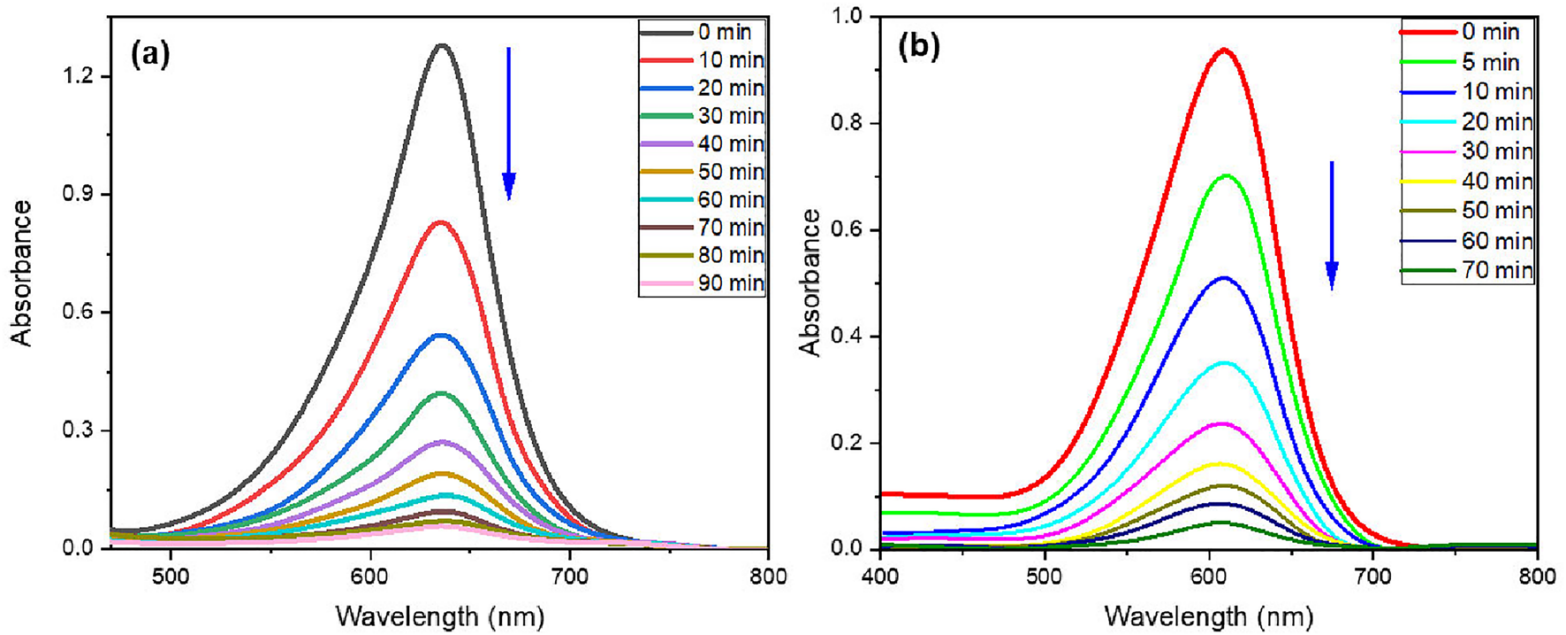
The absorbance of MG (13 mg/L) (**a**) and IC (37 mg/L) (**b**) against time during adsorption onto SFS (20 mg), pH = 7, 25 °C.

This research sets out to develop an efficient adsorbent for the elimination of harmful anionic and cationic dyes that pollute water and endanger human health. As a result, the adsorption abilities of different compositions of spinel ferrite Ag_x_Zn_(0.5-x)_Ni_0.5_Fe_2_O_4_, where x = 0.1, 0.3, and 0.5, to remove MG and IC from simulated wastewater were studied and compared. It was found that Ag_0.1_Zn_0.4_Ni_0.5_Fe_2_O_4_ (SF) is more efficient in dye removal, as shown in Fig. 9. Therefore, Ag_0.1_Zn_0.4_Ni_0.5_Fe_2_O_4_ was chosen for more adsorption studies, especially because it has the highest saturation magnetization for easy separation. Subsequently, the coating of the chosen spinel ferrite (x = 0.1) with a silica layer increased in surface area from 69.79 to 180.08 m^2^/g, as proven by BET. As shown in Fig. 10, the absorbance of MG and IC dyes diminishes over time after adsorption by SFS.

#### The influence of experimental conditions

Significant effects of initial dye concentration, nanocomposite quantity, temperature, and solution pH on the adsorption of various dyes by spinel ferrite@mSiO_2_ (SFS) were observed. The results of varying each variable were examined carefully.

##### Effect of adsorbent dosage

**Figure 11:**
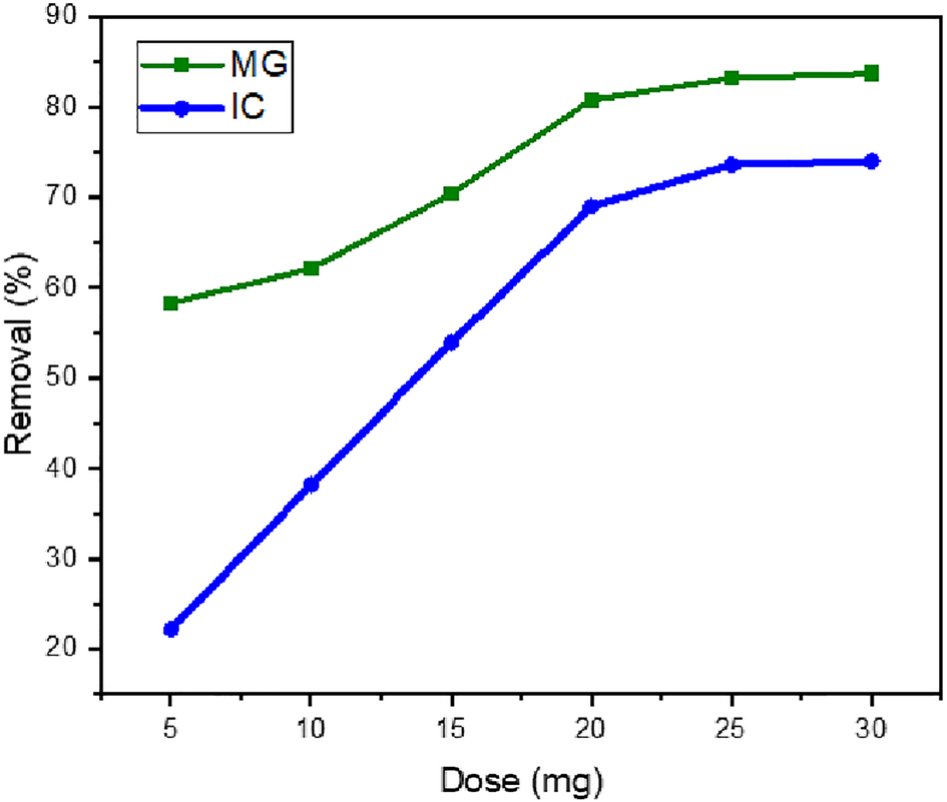
Removal efficiencies of MG (13 mg/L) and IC (37 mg/L) using SFS as a function of adsorbent dosage.

The effectiveness of SFS in eliminating MG and IC was studied by adjusting the dosage from 5 to 30 mg. As illustrated in Fig. 11, the removal percentage for dyes increases as the number of adsorbents increases. This is primarily due to the greater availability of adsorption sites on the adsorbent surface, which allows for more effective interaction between the dye molecules and the adsorption sites^65–67^.

##### The pH effect

**Figure 12:**
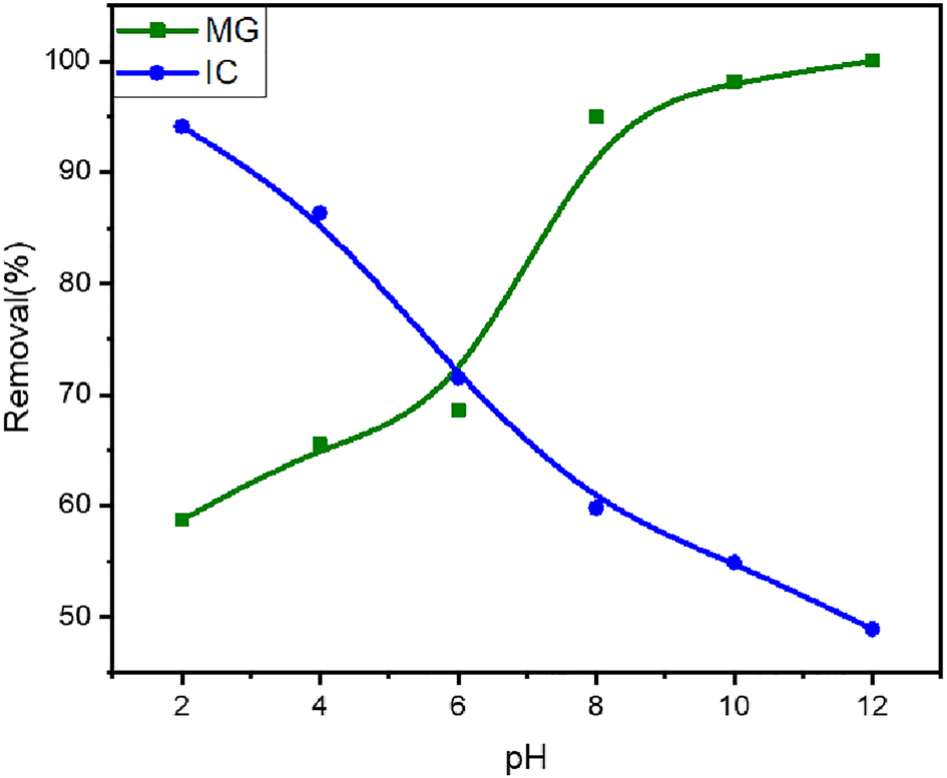
The removal efficiency of MG (13 mg/L) and IC (37 mg/L) utilizing SFS as a function of medium pH molecules are now blocking the active sites of the remaining molecules in solution^75^.

The pH level plays a crucial role in influencing the adsorption process. Both adsorbate dyes (MG or IC) and absorbent SFS possess various surface functional groups that gain or lose protons (H^+^) in response to the pH of the medium^68^. To optimize the adsorption process for pollutant removal, the adsorption process for MG and IC was examined across the pH range (2–12), as shown in Fig. 12.

Pzc measurements show that the point of zero charge (pH_pzc_) of SFS is 6.45. At a pH > 6.45, SFS's surface turns negatively charged due to the ionization of H^+^ from the active groups leaving negative charges; as a result, MG removal efficiencies increase with increasing pH above 6.45, where they reached 96.6% at a pH of 10 due to electrostatic interactions between cationic methyl green and negatively charged adsorbents^69^.

However, IC is negatively charged at low pH and has a pKa of 12.6^70,71^. So, the removal efficiencies of IC decrease at pH > 6.45 due to the increased electrostatic repulsion with negatively charged adsorbent surfaces. This result agrees with the work of Yazdi et al.^72^. As a result, the removal efficiency of IC increases with decreasing pH value until it reaches 93.8% at pH = 2^73^ (Fig. 12), due to the electrostatic attraction between the cation surface of spinel ferrite and the negative IC, and furthermore, the formation of a hydrogen bond. As a result, the alkaline medium was the right choice for removing methyl green dye, and the acidic medium is efficient for removing IC.

##### Effect of the initial concentration of dye

**Figure 13:**
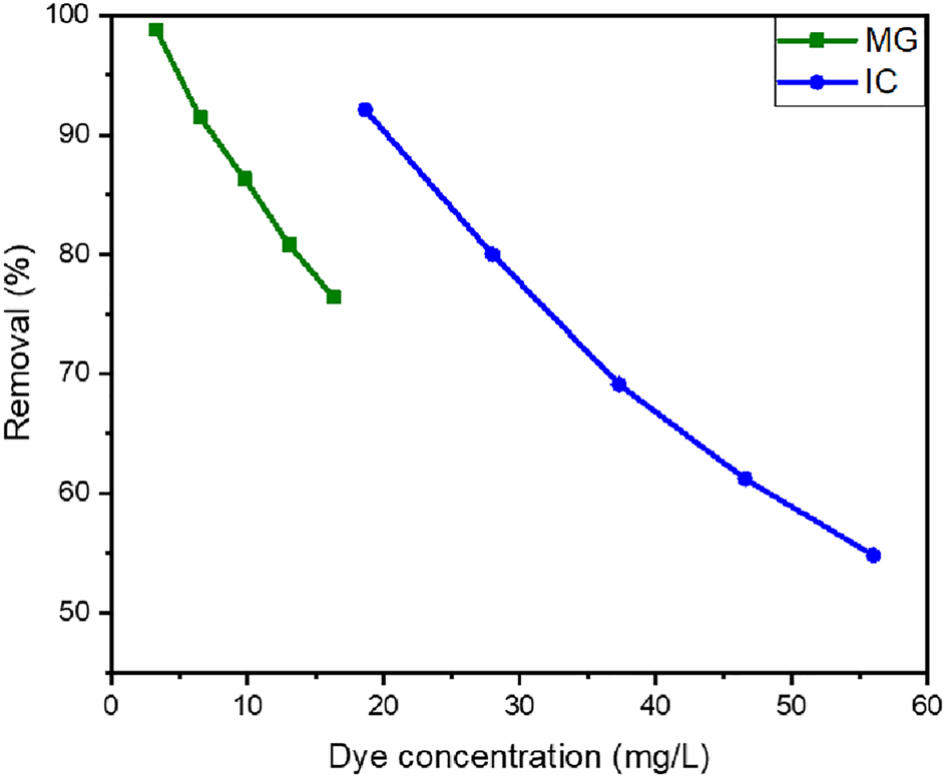
The removal efficiency of MG (3.2–16.3 mg/L) and IC (18.6–56 mg/L) utilizing SFS as a function of initial dye concentration.

The influence of dye concentration on the removal efficiency of pollutant dyes was investigated, as shown in Fig. 13. The removal percentages of MG and IC reached 91% within 90 min below the concentrations of 7 and 19 mg/L for MG and IC, respectively. As the dye concentration increases within 90 min, the percentage of MG and IC that is removed decreases. With an increase in dye concentration, the availability of adsorption sites became restricted, resulting in a decrease in dye removal prior to achieving equilibrium^74^. At higher concentrations, however, the removal effectiveness noticeably drops as a larger number of dye molecules compete for a smaller number of adsorption sites. This decrease is because the previously adsorbed dye molecules are now blocking the active sites of the remaining molecules in solution^75^.

#### Kinetics of adsorption

**Figure 14:**
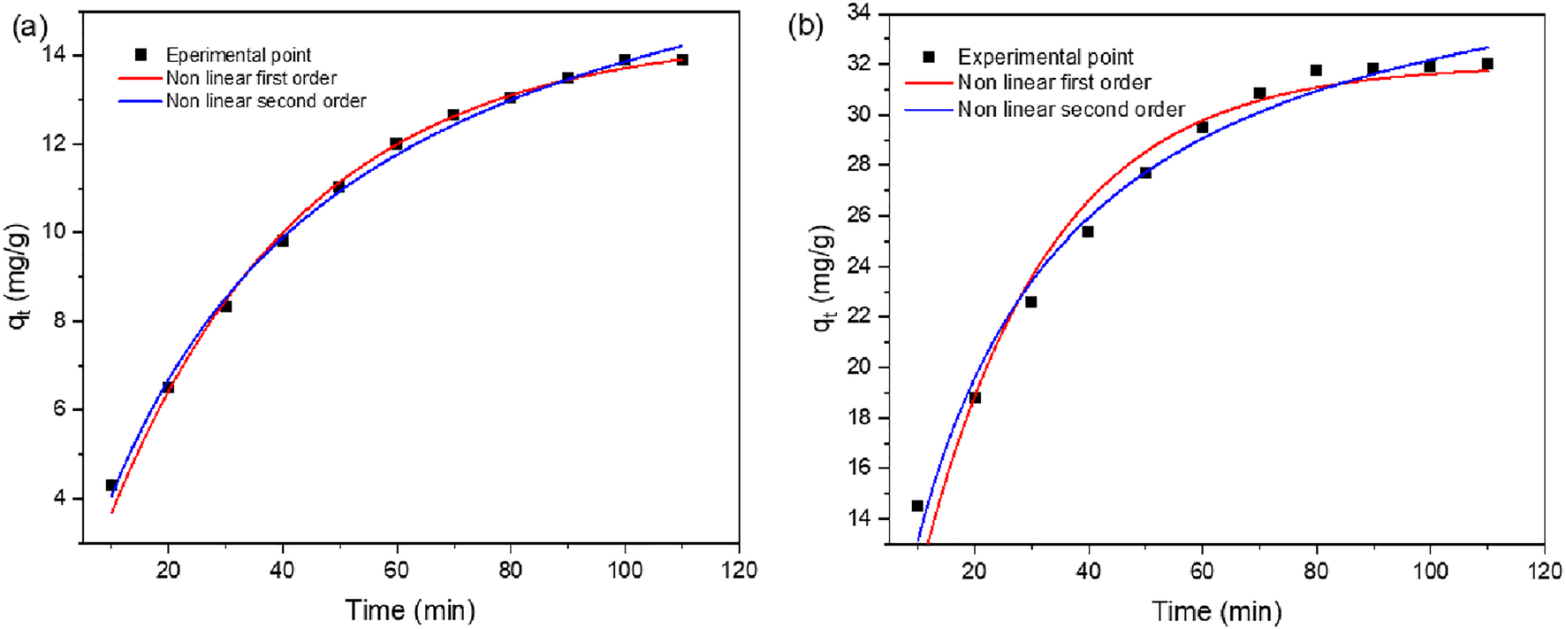
Non-linear kinetic model for adsorption of MG (**a**) and IC (**b**) on SFS.

The time-dependent behaviour of adsorption is depicted by the linear and non-linear adsorption kinetics of the pseudo-first and second-order models in, Eqs. (5)–(8)^76^. For the water treatment to be successful and economical, the adsorption process must be completed quickly. The contact time is the duration of time it takes for the maximum amount of dye adsorbed to reach equilibrium with the adsorbent surface in an adsorption experiment. Figure 14 shows how the quantity of MG and IC adsorbed (q_t_) varies with contact time in a nonlinear kinetic model. After 110 min of interaction, the q_t_ had climbed to an equilibrium level. Also, the mechanism of adsorption of MG or IC dyes onto synthesized ferrite is determined by evaluating the adsorption kinetics in the intra-particle diffusion model, Eq. (9)^77^.

5 $$ln\left({q}_{e}-{q}_{t}\right)=ln{q}_{e}-{k}_{1}t$$

6 $${q}_{t}={q}_{e}\left(1-{e}^{-{k}_{1}t}\right)$$

7 $$\frac{t}{{q}_{t}}=\frac{1}{{k}_{2}{q}_{e}^{2}}+\frac{1}{{q}_{e}}t$$

8 $${q}_{t}=\frac{{k}_{2}{q}_{e}^{2}t}{1+{q}_{e}{k}_{2}t}$$

9 $$ q_{t} = k_{i} t^{1/2} + C $$

**Figure 15:**
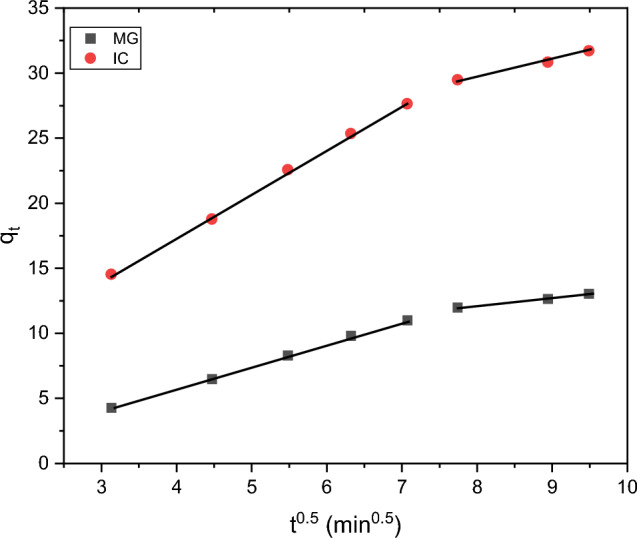
Intra-particle diffusion kinetics model of the of MG and IC on SFS.

Adsorbed dye amounts are given in terms of mg/g at equilibrium (q_e_) and contact time (q_t_) in minutes. Pseudo-first order diffusion has a rate constant of k_1_ (min^−1^), pseudo-second order diffusion has a rate constant of k_2_ (g/mg min), and intra-particle diffusion has a rate constant of k_i_ (mg/g min^1/2^). The values of the correlation coefficient (R^2^) in Table 5, indicate that the linear and nonlinear equations forms of the pseudo-second-order approach, Eqs. (7, 8), respectively, are a better fit and more suited for the experimental data than the linear and nonlinear pseudo-first-order model Eqs. (5, 6), respectively. Also, for each dye under examination, the q_e, exp_, and the q_e_ estimated from the pseudo-second-order model are all quite close to one another, within the experimental errors. Adsorption kinetics may be altered by a number of different processes. Most constrained are the processes of diffusion, which can only occur via (a) extracellular diffusion, (b) boundary layer diffusion, and (c) intra-particle diffusion^77,78^. Therefore, an intra-particle diffusion kinetic model is used to further evaluate the adsorption data and forecast the rate-limiting stage in the process. If the qt vs. t^0.5^ linear plot is through the origin, then intra-particle diffusion is supposed to be in charge of the adsorption process, as predicted by this theory. Figure 15 shows that q_t_ is linearly related to t^0.5^. Here we see examples of both the outer and inner surface diffusions; the former refers to the surface's exterior while the latter describes its interior. According to Table 6, there is a completely quick step of diffusion from the outside onto the adsorbent surface, followed by a completely sluggish step of diffusion into the inner surface^75^.

**Table 5:** Kinetics data of the linear and non-linear forms of adsorption of methyl green and indigo carmine on SF and SFS.

Adsorbent	Dye	Conc. (mg/L)	Adsorption kinetics						
			Pseudo 1st order			Pseudo 2nd order			q_e. exp_ (mg/g)
			q_e. cal_ (mg/g)	k_1_ (min^−1^)	R^2^	q_e.cal_ (mg/g)	k_2_ (g/mg min)	R^2^	
SFS	Linear form								
	MG dye	13	30.7	0.0545	0.963	20.4	0.0014	0.997	23.2
	IC dye	37	50.4	0.0493	0.971	43.4	0.0012	0.999	32.1
	Nonlinear form								
	MG dye	13	14.5	0.029	0.994	19.9	0.0013	0.999	23.2
	IC dye	37	31.9	0.044	0.955	38.9	0.0012	0.991	32.1
SF	Linear form								
	MG dye	13	23.03	0.0427	0.974	20.1	0.0006	0.996	17.8
	IC dye	37	48.9	0.0488	0.925	36.2	0.0008	0.994	27.3
	Nonlinear form								
	MG dye	13	13.6	0.024	0.992	19.1	0.0009	0.997	17.8
	IC dye	37	28.3	0.033	0.994	37.2	0.0008	0.998	27.3

**Table 6:** Adsorption of MG and IC dyes onto SF and SFS through intra-particle diffusion.

Adsorbent	Dye	C_1_ (mg/g)	K_p1_ (g/mg min^1/2^)	R^2^	C_2_ (mg/g)	K_p2_ (g/mg min^1/2^)	R^2^
SFS	MG	1.14	1.72	0.999	7.40	0.59	0.991
	IC	3.91	3.37	0.998	19.7	1.25	0.988
SF	MG	1.75	2.69	0.999	6.01	0.59	0.997
	IC	3.34	0.75	0.998	14.5	1.30	0.986

#### Adsorption isotherm models

**Figure 16:**
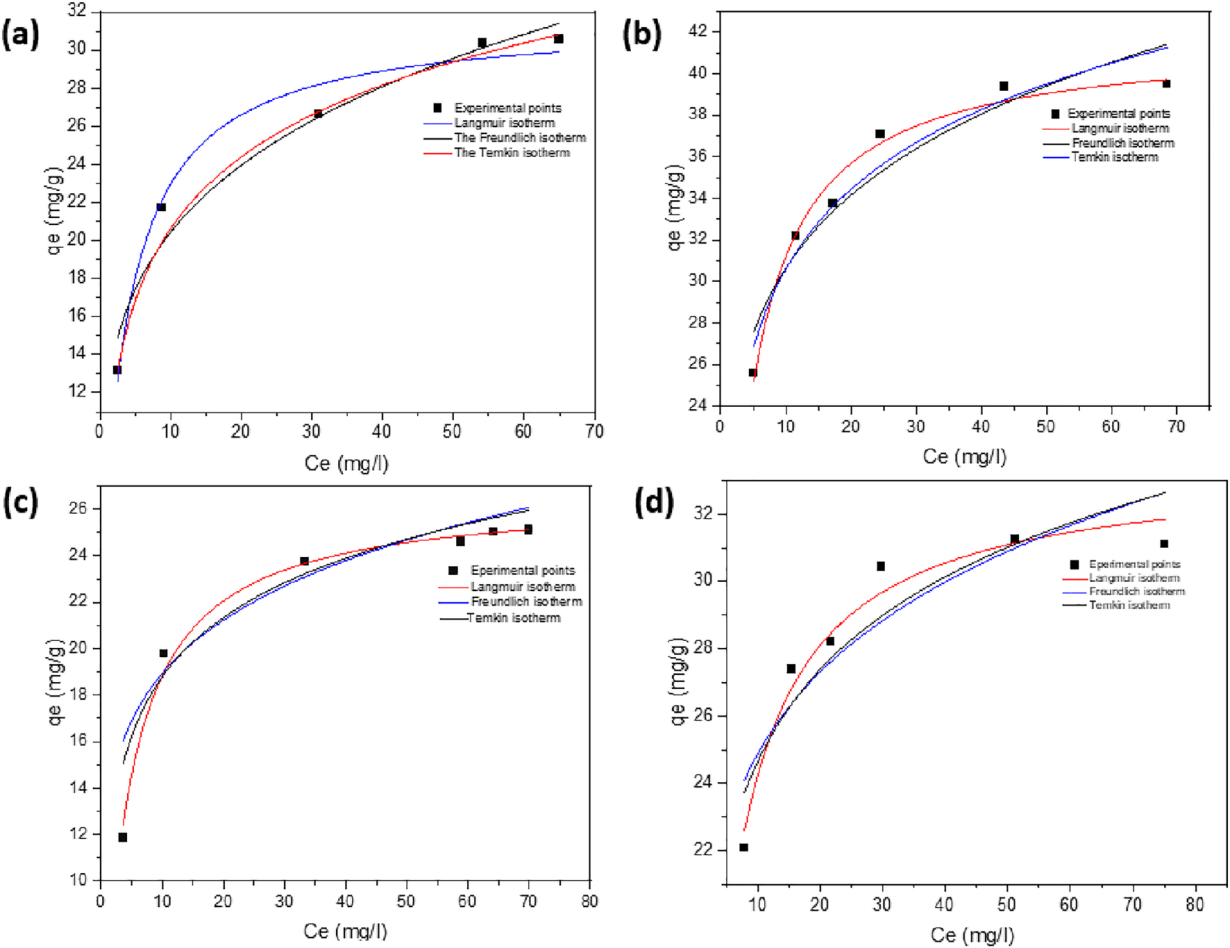
Nonlinear adsorption isotherm of MG (**a**,**b**) and IC (**c**,**d**) on SF and SFS, respectively.

The surface properties, maximum capacity of the adsorbent, and adsorption mechanism were all stated by the values of certain parameters determined from adsorption isotherm models used to examine the produced ferrite's potency and capacity as an adsorbent. Different adsorption isotherm models were employed to compare the results; these included the Langmuir, Freundlich, Temkin, and Dubinin–Radushkevich (D–R) models. Figure 16 shows nonlinear adsorption isotherm of MG and IC on SFS as a compared with SF adsorbent.

Adsorption of dye molecules onto an adsorbent surface has the same activation energy according to the Langmuir isotherm, as shown in Fig. 16, which assumes monolayer adsorption above a homogenous adsorbent surface with a set number of identical sites^79^. This led to the adsorption data of MG and IC being fitted into the nonlinear form of Langmuir's equation^80^, the determination and quantification of the maximum adsorption capacity (q_max_) were performed and tabulated in Table 7.

10 $${q}_{e}=\frac{{q}_{max}.{C}_{e}.{K}_{L}}{1+{C}_{e}.{K}_{L}}$$

where C_e_ is the concentration of dye at equilibrium in mg/l, q_e_ is the adsorption capacity of dye at equilibrium in mg/g, q_max_ is the maximum possible adsorption capacity in mg/g, and K_L_ is the Langmuir constant in L/mg.

**Table 7:** Adsorption isotherms parameters of methyl green and indigo carmine dye at 25°C.

Adsorbent	Adsorbate	Adsorption isotherms								
		Langmuir			Freundlich			Temkin		
		q_max_ (mg/g)	K_L_ (L/mg)	R^2^	n	K_F_ (mg/g(mg/L)^−1/nF^)	R^2^	B (J mol^−1)^	K_t_ (L/mg)	R^2^
SF	MG dye	26.5	0.24	0.995	7.06	12.6	0.991	3.68	16.3	0.944
	IC dye	33.1	0.26	0.981	7.41	18.22	0.821	3.96	49.6	0.814
SFS	MG dye	32.3	0.26	0.991	4.35	12.03	0.691	5.45	4.38	0.99
	IC dye	41.7	0.29	0.988	6.39	21.38	0.881	5.53	25.3	0.92

Table 7 shows the results. Adsorption results agreed with the Langmuir model, which postulated the existence of homogenous adsorption sites on SFS substrate, as indicated by high values of the correlation coefficient (R^2^) for the isotherm plots. The IC dye has sulfonated, amine, and hydroxyl groups in its structure that support electrostatic binding with mesoporous silicate Si–OH through hydrogen bonds, which may explain why the IC dye has a higher Langmuir monolayer coverage capacity value (q_max_) than the MG dye and why the value of the same dye increases when using SFS as an absorbent. The results show that ultimate capacity q_max_ and K_L_ values increased as the temperature of adsorption increased, thus, the affinity of the adsorbent to the investigated dyes increased with increasing temperature.

The maximum monolayer adsorption capacity (q_max_) for methyl green and indigo carmine adsorption is compared across a variety of adsorbent surfaces in Table 8. We found that our SFS as an adsorbent is effective in removing methyl green and indigo carmine from aqueous solutions, when compared to data from the relevant literature.

**Table 8:** Maximum adsorption capacity (q_max_) comparison of MG and IC on various adsorbents.

Dye	Adsorbents	q_max_ (mg/g)	References
IC	Fixed bed activated carbon	25.13	^86^
	Chitosan-graphene oxide	29.7	^87^
	Activated carbon-based KOH	13.4	^88^
	magnesium oxide	0.44	^89^
	TiO_2_/UV/O_2_	14.2	^90^
	SF	33.1	The current study
	SFS	41.7	The current study
MG	Polyaniline coated Silica gel	7.65	^91^
	GO nanosheets	4.76	^92^
	Amberlite XAD-4 resin	1.157	^93^
	Sepiolite	0.094	^94^
	SF	26.5	The current study
	SFS	32.3	The current study

An experimental expression that accounts for surface heterogeneity and an exponential distribution of site and energy energies is the Freundlich isotherm^81^. The Freundlich adsorption Eq. (11) describes this equilibrium.

11 $${q}_{e}={K}_{f}.{{C}_{e}}^{1/n}$$

The Freundlich isotherm constant is denoted by k_F_ ((mg/g) (mg/L)^−1/n^_F_)^82^, where n is the dimensionless exponent of the Freundlich. K_F_ and n are the determined values. Estimates of n_F_ in this work ranged from 4 to 7, indicating that the adsorption processes are close to irreversible^83^. When the K_F_ value is large, the adsorption is driven with greater efficiency. This means that the adsorption driving force for IC dye is greater than that for MG dye, and the driving force grows when SFS is employed as the adsorbent. The favourable nature of the adsorption process is confirmed at higher temperatures by an increase in K_F_ value^84^.

Adsorption data for MG and IC were analyzed using the Temkin isotherm model. This model supports limited interactions between the adsorbent and adsorbate and demonstrates that all molecules in the surface layer have decreased adsorption energies at the cover surface. Additionally, it was believed that adsorbate-adsorbent interactions directly reduced the adsorption heat with coverage^85^. Equation (12) represents the Temkin isotherm model.

12 $${q}_{e}=\frac{RT}{b}.ln{K}_{T}+\frac{RT}{b}.ln{C}_{e}$$

where K_T_ (L/mol) represents the equilibrium binding constant, R is the perfect gas constant, T is the absolute temperature, and b (kJ/mol) represents the heat of adsorption. Table 7's b values match up with an adsorption mechanism involving electrostatic interactions and hydrogen bond formation. Temperature has a beneficial influence on the binding energy of dyes with ferrite surfaces, as evidenced by the rise in K_T_ as the temperature rises.

To identify the adsorption mechanism (physical or chemical), we can use Dubinin–Radushkevich isotherm Eqs. (13–15) to determine the activation energy E (KJ/mol). Adsorption has been explained by ion exchange adsorption if E ranges from 8 to 16 kJ/mol; if E is 8, the process has been validated physically. Adsorption in this study may occur by chemisorption or ion exchange, where E ranges from 8 to 16 kJ/mol.

13 $$ln{q}_{e}=ln{q}_{s}-\beta {\varepsilon }^{2}$$

where q_s_ (mol/kg) is the theoretical capacity of adsorption calculated from Eq. (13) of the D-R model, ε (kJ/mol) is the Polanyi potential, and β (mol^2^/kJ^2^) is the mean free energy of adsorption for each molecule adsorbed as given by Eq. (14).

14 $$E=\frac{1}{\sqrt{2\beta }}$$

15 $$\varepsilon =RTln\left(1+\frac{1}{{C}_{e}}\right)$$

#### Thermodynamics studies

**Figure 17:**
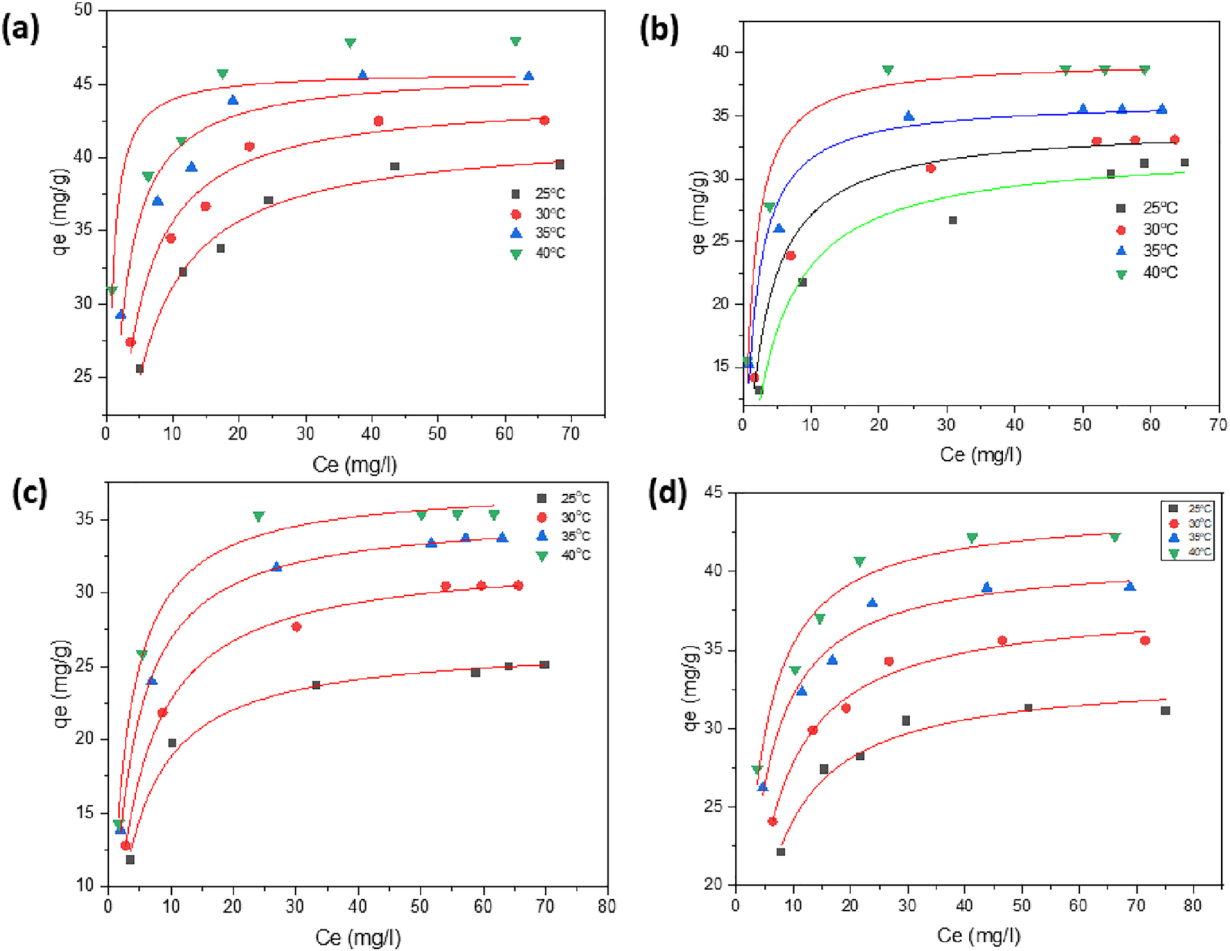
Nonlinear Langmuir adsorption isotherm of MG (**a**,**b**) and IC (**c**,**d**) on SF and SFS, respectively at different temperatures.

The Langmuir model is the best-fitted model at the different temperatures according to the R^2^ values in Table 7. The effect of temperature on the adsorption process according to the Langmuir isotherm is studied as shown in Fig. 17, where q_max_ and K_L_ (L/mg) at different temperatures are shown in Table 9. To determine the thermodynamic parameters, the obtained equilibrium constant must become dimensionless before being applied to the Vant´Hoff equation using Eq. 16^95^.

16 $${k}_{e}^{0}= (1000.{K}_{L}.mol.wt\, of\, dye).\frac{\left[Dye\right]^\circ }{{\varvec{\gamma}}}$$

where γ is the activity coefficient (dimensionless) and [Dye]° is the standard concentration of dye (1 mol/L).

The entropy ΔS° and enthalpy ΔH° of adsorption are determined according to the Eqs. (17, 18) from the intercept and slope of the relationship between $ln{K}_{e}^{0}$ vs. 1000/T, respectively, where R is the perfect gas constant (8.314 J/mol K), and T is the absolute temperature^96^. The adsorption of MG or IC dye onto the synthesized ferrite is endothermic due to the positive values of ΔH° as shown in Table 9. Also, the measured ΔH° values varied from 25 to 48 kJ/mol, suggesting that both physisorption and chemisorption may be involved in the adsorption of the dyes of interest^97^. Adsorption is characterized by an increase in the degree of disorder at the solid-solution interface, as measured by an increase in the entropy parameter, ΔS°^98^. This is the normal outcome of electrostatic force interactions that lead to the phenomenon known as physical adsorption. The spontaneous process for MG and IC sorption is revealed by negative values of ΔG°, and as the temperature is raised, the value of ΔG° decreases even further, showing that the adsorption process is more favoured at a higher temperature^99^**.**

**Table 9:** Adsorption process thermodynamic characteristics for both MG and IC at various temperatures.

Adsorbent	DYE	MG				IC			
	Temp. (K)	298	303	308	313	298	303	308	313
SF	q_max_ (mg/g)	26.5	32.57	35.3	37.3	33.1	38.4	40.6	43.5
	K_L_ (L/mg)	0.24	0.26	0.34	0.41	0.26	0.273	0.36	0.41
	R^2^	0.991	0.998	0.997	0.998	0.987	0.976	0.966	0.963
	K_L_ (L/mol)	1.56 × 10^5^	1.7 × 10^5^	2.22 × 10^5^	2.68 × 10^5^	1.21 × 10^5^	1.27 × 10^5^	1.67 × 10^5^	1.91 × 10^5^
	K_e_^0^	1.57 × 10^5^	1.7 × 10^5^	2.22 × 10^5^	2.68 × 10^5^	1.21 × 10^5^	1.27 × 10^5^	1.67 × 10^5^	1.91 × 10^5^
	ΔG° (KJ/mol)	29.5-	− 30.5	− 31.5	− 32.4	− 28.8	− 29.7	− 30.7	− 31.6
	ΔH° (J/mol)	28.96				25.4			
	ΔS° (J/mol K)	196.2				182.07			
	R^2^	0.96				0.936			
	R^2^_adj_	0.94				0.91			
SFS	q_max_ (mg/g)	32.3	34.27	36.24	37.9	41.7	44.2	45.8	50.9
	K_L_ (L/mg)	0.26	0.38	0.67	0.87	0.29	0.41	0.71	0.91
	R^2^	0.987	0.992	0.928	0.828	0.995	0.993	0.991	0.992
	K_L_ (L/mol)	1.57 × 10^5^	2.48 × 10^5^	4.37 × 10^5^	5.68 × 10^5^	1.35 × 10^5^	1.91 × 10^5^	3.31 × 10^5^	4.24 × 10^5^
	K_e_^0^	1.57 × 10^5^	2.48 × 10^5^	4.37 × 10^5^	5.68 × 10^5^	1.35 × 10^5^	1.91 × 10^5^	3.31 × 10^5^	4.24 × 10^5^
	ΔG° (KJ/mol)	− 29.6	− 31.3	− 32.9	− 34.6	− 29.2	− 30.7	− 32.32	− 33.85
	ΔH° (J/mol)	48.7				41.6			
	ΔS° (J/mol K)	330				305.1			
	R^2^	0.984				0.983			
	R^2^_adj_	0.976				0.974			

17 $$ln{K^\circ }_{e}=\frac{{\Delta S}^{^\circ }}{R}-\frac{{\Delta H}^{^\circ }}{RT}$$

18 $$\Delta {G}^{^\circ }= {\Delta H}^{^\circ }-T{\Delta S}^{^\circ }$$

#### Characterization Silica coated spinel ferrite after Adsorption.

##### FTIR analysis

**Figure 18:**
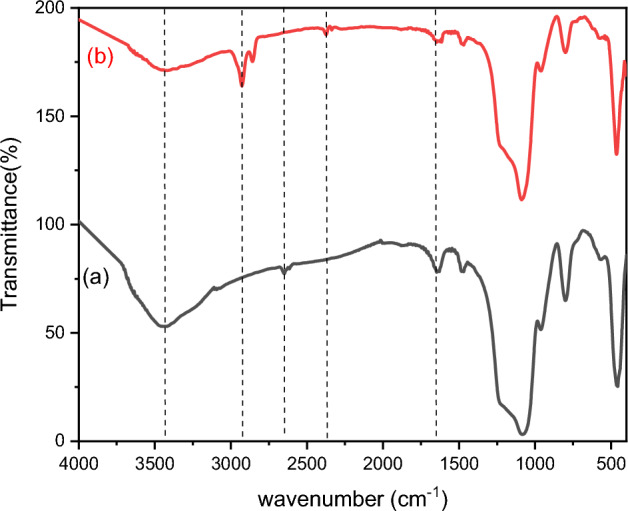
FTIR of SFS before (**a**) and after adsorption (**b**) of IC.

A Comparison of the FTIR spectra of the used SFS before and after adsorption of IC as a model of the investigated dye showed the appearance of additional peaks after their contact with the IC dye (Fig. 18). Peaks appearing at 2930 cm were due to CH stretching, at 1374 cm^−1^, it corresponds to the C–H bending vibration or C–N stretching vibration of amines^100^. A comparison between newly appeared peaks and those of IC showed that the new peaks were probably the characteristic of this textile pollutant. The band broadening about 3400 cm^−1^ is assigned to the vibration formed by hydrogen-bonded –O–H groups in the absorbent and adsorbate molecules. The band broadening at 3390, 3433 cm^−1^ indicate to the hydrogen bonding in comparison to the FTIR analysis before the adsorption process as well as existence of (-NH) stretching bond of dye^101^.

The N–H bond between the dye's N atom and the H–O–H bond resulted in a noticeable shift of the H–O–H peak from 1638 to 1627 and an increase in its width. This effect resulted from the establishment of oxygen-hydrogen bonds between the dye and the adsorbent^102^. The adsorption of molecules onto the SFS nanoparticles in this study is consistent with physical adsorption mechanisms, involving hydrogen bonding and electrostatic forces. The low adsorption heat indicated by the Temkin isotherm model, the formation of a monomolecular layer according to the Langmuir isotherm, and FTIR analysis collectively support the physical adsorption process on the SFS nanoparticle surface.

##### SEM/EDX analysis

**Figure 19:**
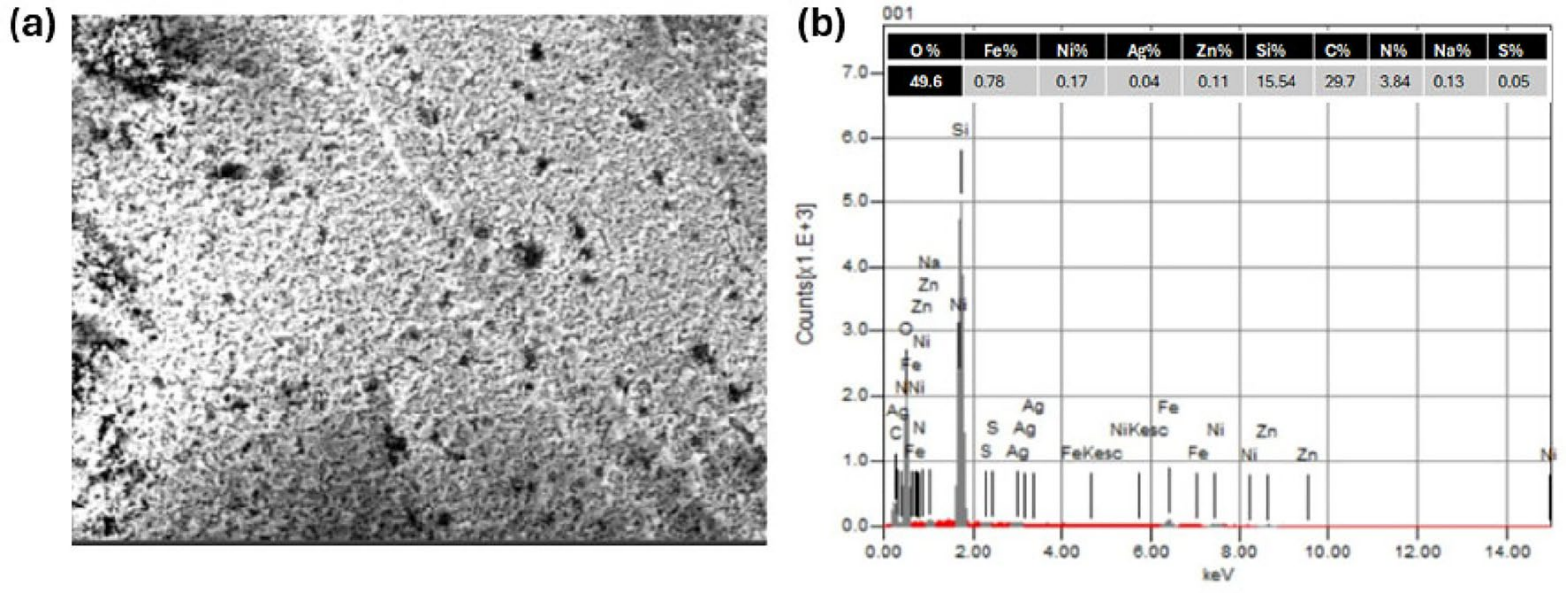
SEM/EDX of SFS after adsorption of IC.

Scanning electron microscopy revealed a substantial change in surface shape following adsorption (Fig. 19). SEM–EDX analysis was used to analyse the morphological aspects of SFS. The dye molecules appear on the surface of spinel ferrite. The principal components of ferrite/IC are Fe, Ni, Zn, O, Si, and C, N from dyes, according to the EDX analysis, confirming the efficient adsorption of IC.

#### Reusability and recovery study

**Figure 20:**
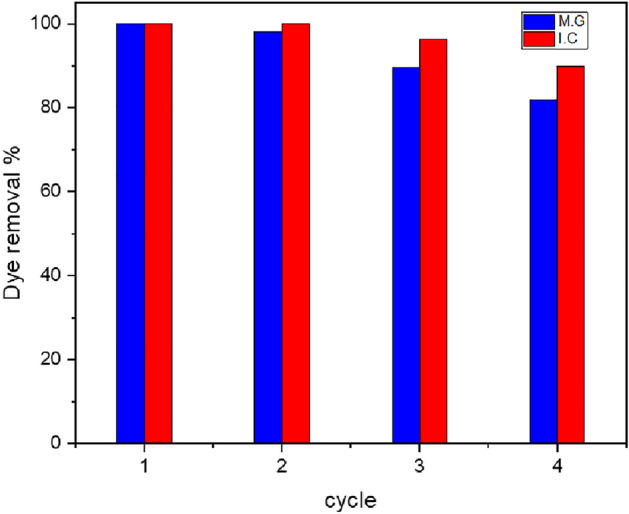
The reuse of dyed SFS samples.

The regeneration and reusability of adsorbent surfaces are critical aspects of determining their true value. The use of adsorbents with high adsorption capacity and high desorption assets reduces additional environmental pollution and overall costs. As a result, desorption studies on SFS are carried out to determine its recyclable accessibility. The adsorbent was recovered by treating the used sample with 0.1 M HCl for 2 h, washing it three times with distilled water, and finally testing the filtrate with a silver nitrate solution to make sure the HCl was gone before drying it at 45 °C for 18 h. For four cycles, 20 mg of adsorbent was used. The elimination percentage changed with each cycle, as seen in Fig. 20^103^.

## The proposed mechanism of adsorption

**Figure 21:**
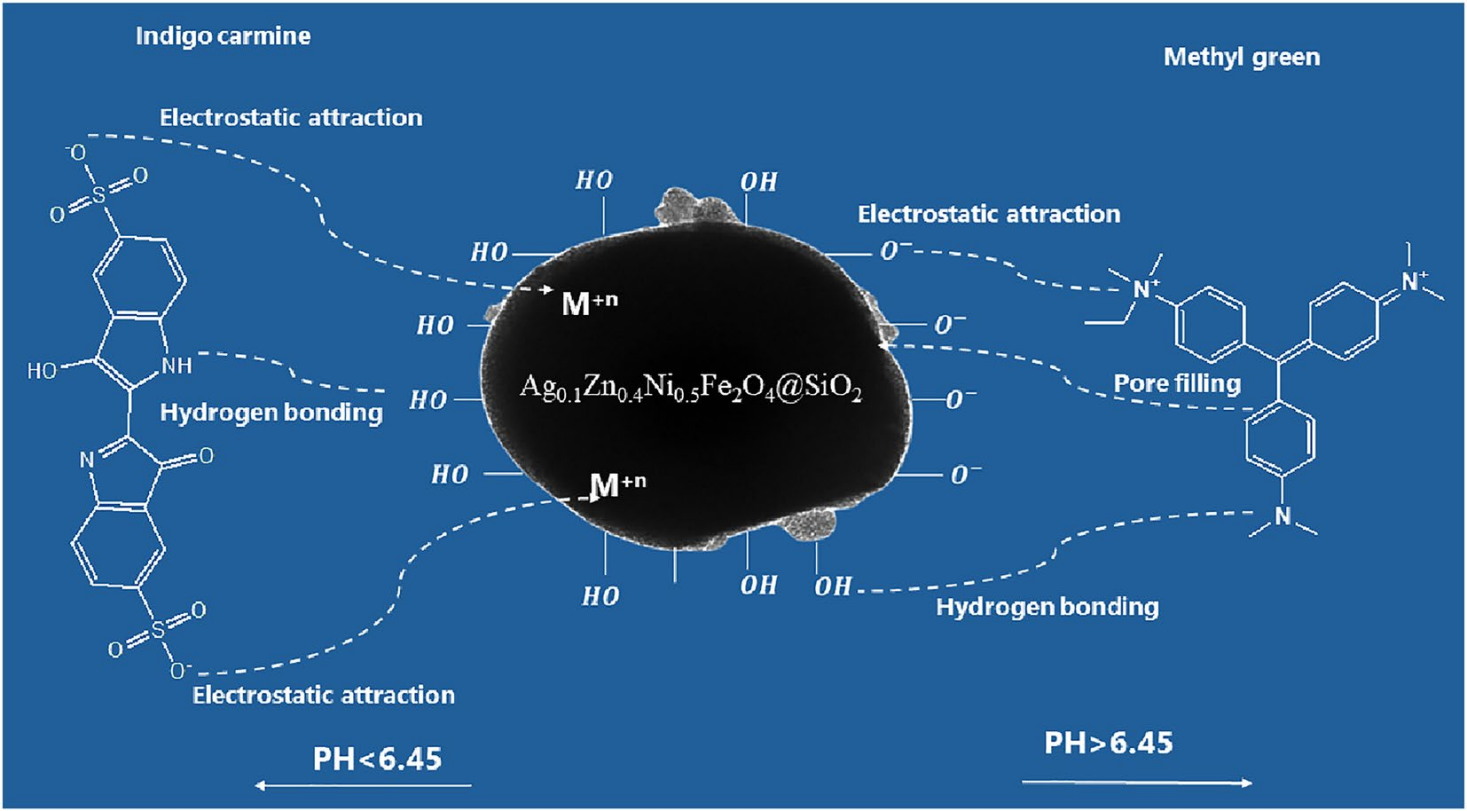
Proposed mechanism of the adsorption of MG and IC dyes on SFS.

Based on the provided information and the characteristics of the adsorption process, here is a suggested mechanism for the adsorption of MG and IC dyes onto the synthesized ferrite material. From pH and PZC studies, when the pH level goes above 6.45, active groups ionize and make the surface of the synthesised SFS material negatively charged. This negative charge makes it easier for the positively charged cationic methyl green (MG) dye molecules to stick to the negatively charged surface of the adsorbent. This electrostatic interaction is a dominant driving force for MG adsorption. On the other hand, indigo carmine (IC) dye is negatively charged at low pH. The IC dye contains sulfonated, amine, and hydroxyl groups in its structure. These functional groups can form hydrogen bonds with the mesoporous silicate (Si–OH) on the adsorbent surface. The presence of hydrogen bonding sites enhances the adsorption of IC dye onto the adsorbent. All these were confirmed by Temkin, Freundlich, and Dubinin-Radushkevich. It is thought that an increase in temperature gives more thermal energy, which helps both MG and IC dyes stick to the adsorbent surface because the adsorption process is endothermic. This increased energy facilitates the interactions between the dye molecules and the adsorbent. The positive values of ΔH°, which range from 25 to 48 kJ/mol, suggest that MG and IC dyes may bind by both physisorption and chemisorption mechanisms, using hydrogen bonds and electrostatic interactions. The second order and intra-particle diffusion kinetics indicate that the adsorption process involves a rapid initial step of diffusion from the external solution to the adsorbent surface, followed by a slower step of diffusion into the inner surface of the adsorbent as shown in Fig. 21.

## Conclusion

Spinel ferrite@SiO_2_ (SFS) is a powerful adsorbent for cleaning textile processing wastewater containing cationic and anionic dyes. This assertion has been supported by the high surface area provided by the coated silica and the ease of separation provided by the magnetite core. The adsorptive activity of SFS against pollutant dyes is due to electrostatic interactions and hydrogen bond formation via Si–OH and Si=O, including wrinkles, flaws, and residual functional groups. Notably, the adsorption efficiency for anionic dye removal is significantly higher than that for cationic dye. Therefore, the adsorbate's ionic characteristics and the nature of the adsorbent's active sites determine the adsorption mechanism. As an anionic dye, IC's molecular structure includes –SO^3−^, OH^−^, and NH^−^ groups, all of which are capable of interacting with the absorbent. The aforementioned energetic sites explain why they are so effective at attracting ICs. MG, a cationic dye, should have a lower adsorption capability since it contains a positive canter and a smaller active group capable of forming hydrogen bonds than IC.

It is speculated that the ionic nature of both MG and IC is responsible for their adsorption onto the SFS. Langmuir isotherm model fitting confirmed the development of a dye monolayer across the adsorbent surface. The dramatic rise in q_e, cal_ is reflected in the q_max_ that was attained. Since physisorption results in lower free energy values ΔG°, it is the adsorption mechanism of choice.

### Ethical approval

No ethical issues were violated in this study.

### Consent to participate

All the authors agreed to participate in this work.

## Data Availability

All data generated or analyzed during this study are included in this article.
